# On-site evaluation as external quality assessment of microbiology laboratories involved in sentinel laboratory-based antimicrobial resistance surveillance network in Burkina Faso

**DOI:** 10.1186/s13756-023-01362-5

**Published:** 2024-01-08

**Authors:** Emmanuel Zongo, Emilie Dama, Dame Yenyetou, Merci Muhigwa, Abdoulaye Nikiema, Georges Anicet Dahourou, Abdoul-Salam Ouedraogo

**Affiliations:** 1 Higher Institute of Health Sciences, Nazi BONI University, Bobo-Dioulasso, Burkina Faso; 2 National Reference Laboratory for Antimicrobial Resistance, Souro SANOU University Hospital Center, Bobo-Dioulasso, Burkina Faso; 3 Laboratory of Emerging and Re-emerging Pathogens (LaPathER), Doctoral School of Health Sciences, Nazi BONI University, Bobo-Dioulasso, Burkina Faso; 4 Health Sciences Research Institute, Ouagadougou, Burkina Faso; 5 US Centers for Disease Control and Prevention, Ouagadougou, Burkina Faso; 6 Integrated Quality Laboratory Services, Ouagadougou, Burkina Faso; 7 MURAZ Center, Bobo-Dioulasso, Burkina Faso

**Keywords:** AMR, Sentinel laboratories, External quality assessment, Burkina Faso

## Abstract

**Background:**

The laboratory-based surveillance of antimicrobial resistance (AMR) is a priority component of the multisectoral national action plan to combat AMR in Burkina Faso. This study aimed to assess the QMS of microbiology laboratories involved in the Sentinel laboratory-based antimicrobial resistance surveillance network in Burkina Faso.

**Methods:**

A cross-sectional study was conducted from September 1st to November 30^th,^ 2022. The external quality assessment (EQA) method used was on-site evaluation using a checklist that was developed and validated by a technical committee of experts. Teams of two, including an antimicrobial susceptibility test (AST) specialist and a QMS specialist, were trained on this checklist to conduct the assessment. Satisfactory performance was defined as any on-site evaluation score 80% and above with the aim of developing action plans to address gaps.

**Results:**

All 18 laboratories were evaluated. The overall average performance score of the participating laboratories was 40%. The highest overall performance score was 58%, and the lowest overall performance score was 26%. The average overall scores were not significantly different between private and public laboratories (*p* value = 0.78). The only section of the checklist with the satisfactory performance concerned the “Analytical step of AST”, with 76.5% (13/17) of the sentinel laboratories having a score ≥ 80%.

**Conclusion:**

The performance of the QMS of the sentinel laboratories in Burkina Faso for AMR surveillance was unsatisfactory, and a corrective action plan was proposed to support these laboratories in improving their QMS over the next 3 years.

## Background

According to the World Health Organization (WHO), “AMR is one of the top 10 global public health threats facing humanity” [1]. The authorities of Burkina Faso made the fight against antimicrobial resistance (AMR) a priority. Indeed, poor access to portable water and unfavourable hygiene conditions increase the risk of infection and, consequently, the risk of transmission of resistant bacteria. Antibiotic therapy is mainly empirical and based on a syndromic approach, which could lead to the emergence of resistance. Following the recommendations of the WHO, a multisectoral national action plan to combat AMR has been developed and validated since 2017 [2]. The laboratory-based surveillance of antimicrobial resistance is a priority component of this plan [3]. It has been implemented since 2018 through a national network of laboratories called “sentinel laboratories”. A National Reference Laboratory for AMR (NRL-AMR) has also been designated to ensure the technical coordination of the activities. In practice, surveillance consists of the monthly collection of data from antibiotic susceptibility tests routinely carried out in sentinel laboratories and transmitted using WHONET software, the monitoring of resistance observed in sentinel laboratories and the confirmation of the detected resistance phenotypes by the NRL-AMR in accordance with the guidelines and standards operating procedures and manuals designed for this purpose, such as the Laboratory-based AMR surveillance guideline and AST procedures manual [3–4]. These data, once collected, are analyzed at Department of Medical Biology Laboratories (DLBM) each year and allow the updating of the list of essential antibiotics, the national treatment guidelines and the evaluation of the effects of the different treatment strategies put in place. All of this requires accurate and reliable bacterial identification and antimicrobial susceptibility testing (AST) data [5]. Laboratories participating in surveillance should implement a quality management system (QMS) according to national and international quality standards and regulations to ensure the accuracy, reliability and timeliness of reported results [6]. Indeed, when the AST results provided by laboratories are doubtful or lack reliability and precision, the information obtained from the analysis of these data will be erroneous, which will have serious consequences. A study conducted by Dougnon et al. in 2016 in Benin showed that the reference strain *Escherichia coli* ATCC 25,922 was resistant to all the antibiotics tested on three brands of commercially available antibiotic discs, which is contrary to the normative profile of the strain studied [7]. Katawa et al., in 2021 in Lomé, evaluated the steps for performing the AST in 3 laboratories, A, B and C, and found that the concentrations of bacterial suspension were higher than 0.5 McFarland standard for laboratories A and B [8]. Diallo et al. in 2023 in Burkina Faso reported amoxicillin and amoxicillin + clavulanic acid discs that did not meet specifications for amoxicillin or clavulanic acid content, inappropriate storage of antibiotic discs, and a lack of internal quality control (IQC) of antibiotic discs prior to use [9]. A recent review on the contribution of diagnostics in the fight against AMR in West Africa highlighted the need for African laboratories to implement QMS [10]. In 2017, Saeed et al. in Pakistan found low participation of private and public AMR surveillance laboratories in the external quality assurance assessment system [11]. In addition, the implementation of quality assurance and external quality assessment (EQA) is required for the achievement of objective 2 of the global action plan on AMR [12]. Finally, Burkina Faso’s regulatory Good Laboratory Practice Guidelines requires that “Any laboratory performing testing shall have a QMS” [13]. All laboratories designated for AMR surveillance in Burkina Faso shall conform to this requirement and implement a QMS.

This study aimed to assess the quality of microbiology laboratories involved in the Sentinel laboratory-based antimicrobial resistance surveillance network in Burkina Faso to contribute to improving their QMS.

## Methods

### Type and period of the study

A cross-sectional study of the quality approach in sentinel laboratories involved in the Sentinel laboratory-based antimicrobial resistance surveillance network in Burkina Faso was conducted from September 1st to November 30th 2022. The duration of the on-site evaluation was two days per sentinel laboratory and seven days were required for data analysis, report writing and feedback to the participating laboratory.

### Participating laboratories and areas of external quality assessment

**Fig. 1:**
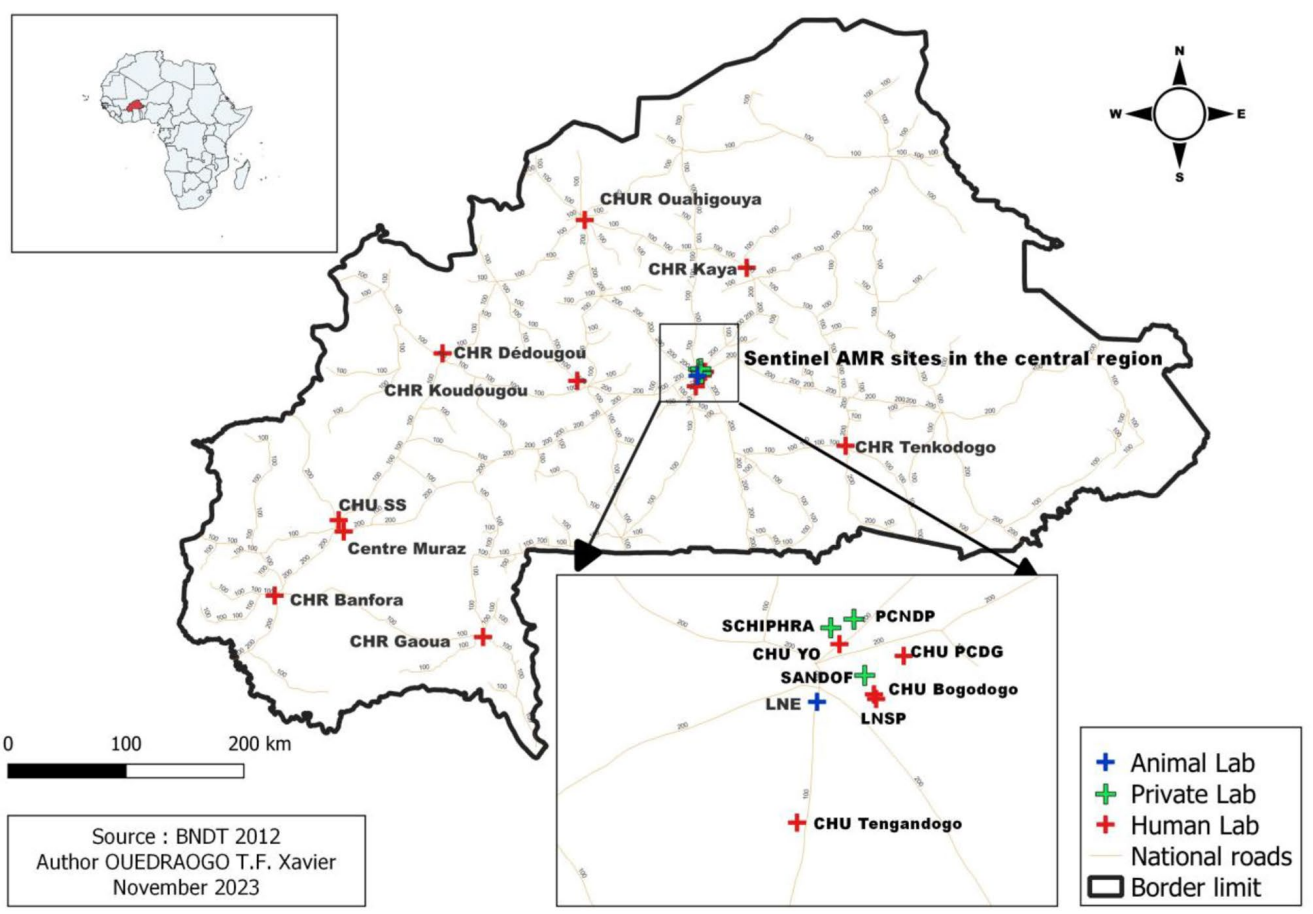
Distribution of AMR sentinel surveillance sites, Burkina Faso

Laboratories are selected on the basis of their capacity in terms of human resources (biomedical technologists and biologists) and equipment to detect resistance effectively using standardised methods, as well as their geographical distribution in order to cover the whole country [3]. There was no minimum number of tests required of sentinel sites, but the criteria established allow to have sites with a high level of activity and a high number of tests. They should be able to identify a list of selected pathogens and perform AST. The methods used are those described in the national procedures, such as coproculture, blood culture, Cytobacteriological examination of urine, culture of pus and puncture fluids, AST using the agar diffusion method or the automated method. The pathogens selected for surveillance are *Escherichia coli* (isolated from blood, urine, stool), *Klebsiella spp* (blood, urine), *Staphylococcus aureus* (blood, pus), *Salmonella spp* (stool and blood), *Shigella spp* (stool), *Pasteurella spp* (sputum), *Streptococcus pneumoniae* (Cerebrospinal fluid, blood), *Pseudomonas aeruginosa* (blood, urine, pus), *Acinetobacter baumanii* (blood), *Neisseria meningitidis* (Cerebrospinal fluid), *Haemophilus influenza* (Cerebrospinal fluid), *Neisseria gonorrhoeae (*Urethral and cervical swabs), *Mycobacterium tuberculosis* in particular for the National Livestock Laboratory (nasopharyngeal secretions, milk). The antibiotics tested depend on the pathogen and are defined in the surveillance guide. These laboratories also detect resistance phenotypes such as extended-spectrum β-lactamase (ESBL), carbapenemases and Methicillin-resistant *Staphylococcus aureus* (MRSA) [3]. Eighteen (18) laboratories, public and private, were expected to take part in this EQA. These laboratories were selected and recognized by the Department of Medical Biology Laboratories (DLBM) as sentinel laboratories for AMR surveillance. Figure 1 shows the distribution of sentinel laboratories in the AMR surveillance network in Burkina Faso.

### Methods and criteria for EQA in sentinel laboratories for AMR surveillance in Burkina Faso

To carry out the EQA in the AMR sentinel laboratories, we carried out an external assessment of the “on-site evaluation” type. A documentary review was carried out on all the specific requirements for AMR surveillance activities, namely, the Standardized Operating Procedures (SOP) for AST, guidelines, checklist and regulatory quality standard. These documents were used to develop the assessment checklist. These guidelines, Manuals, checklists and reference documents used were as follows:

- The regulatory quality standard: Good laboratory practices in Burkina Faso [13];
- Manual of procedures for AST realization in Burkina Faso [4];
- The National Guide for AMR surveillance in laboratory [3];
- The National Guidelines for Biosafety and Biosecurity in medical biology laboratories [14];
- Laboratory Assessment of Antibiotic Resistance Testing Capacity, User’s Guide and Questionnaire. VS 2.0. August 2020 [5];
- The Stepwise Laboratory Improvement Process Toward Accreditation (SLIPTA) checklist [15].

### Development, validation and description of the assessment tool

To assess the QMS of AMR sentinel laboratories, a checklist was developed by a technical committee. This committee was made up of specialists and national experts in the quality approach, SLIPTA/African Society for Laboratory Medicine (ASLM)-certified auditors, medical bacteriologists with extensive experience in the clinical bacteriology laboratory and particularly in carrying out AST and QMS specialists in the biology medical laboratory. The validation of the checklist was done in the presence of all the actors participating in the evaluation, representatives of the DLBM and the head of the NRL-AMR. The approved checklist was an Excel file, which was coded to automatically generate a score. The possible answers to the questions and points were “Yes: 1”, “Partial: 0.5”, “No: 0” and “Not applicable”. The total number of scores obtained per section was summed to give one hundred and sixty-five (165) points. To obtain the proportion per section, the score for the section was divided by the total score, which is one hundred and sixty-five (165), and then multiplied by one hundred (100).

The checklist had thirteen sections, of which eleven sections concerning quality system essentials were scored. Sections are numbered from 1 to 13.

Sections 1 and 2 provide instructions for use and references. For the evaluation of Sect. 13 of the checklist “Analytical step for the AST”, we proceeded with direct observation of all the steps of carrying out the AST, from the preparation of the inoculum to the reading of the inhibition diameters and the interpretation according to the diffusion method in agar medium. Table 1 describes the sections of the assessment checklist.

**Table 1:** Different sections of the checklist for on-site evaluation in sentinel laboratories

N^o^	Section title	Number of possible Points	%
**Section 1**	Instructions for use	-	N/A^a^
**Section 2**	References	-	N/A
**Section 3**	Registration for a quality program and certification	7	4.2
**Section 4**	Documents and records	14	8.5
**Section 5**	Quality assurance	23	13.9
**Section 6**	Material and product	16	9.7
**Section 7**	Internal Quality Control	18	10.9
**Section 8**	Management of staff competencies	11	6.7
**Section 9**	Failure analysis, problem resolution and root cause analysis	4	2.4
**Section 10**	External Quality Assessment (EQA^b^)	11	6.7
**Section 11**	Storage of antibiotic discs	12	7.3
**Section 12**	Biosafety	27	16.4
**Section 13**	Analytical step for the AST^c^	22	13.3
**Overall score**		165	100

^a^Not Applicable; ^b^External Quality Assessment. ^c^Antimicrobial Susceptibility Test

### EQA teams of the sentinel laboratory for AMR surveillance

Four (04) teams were formed to conduct the on-site evaluation. The evaluators were trained on the checklist. Each team consisted of experienced professionals, including a bacteriologist with good experience in antimicrobial resistance in the clinical bacteriology laboratory as well as the steps of carrying out the AST and a QMS specialist in the medical biology laboratory. A supervisor was also assigned to each team to oversee the evaluation. The principles of independence and objectivity as well as the conflicts of interest of evaluators and supervisors were considered in the composition of the teams. The evaluators were also reminded of the professional conduct (good relations with laboratory staff, private discussions with the laboratory manager in case of embarrassing or irritating findings, authorization before taking images, etc.). To facilitate the conduct of the assessment, administrative documents were prepared and approved by the authorities of the Ministry of Health (MoH) of Burkina Faso. The letter of authorization from the health authorities of the MoH also facilitated the participation of the sentinel laboratories. The data sources identified were a review of laboratory documentation, interviews with staff and direct observation.

### Data analysis, performance assessment criteria and ethical considerations

The data from the evaluation tool were analyzed using the pivote table of Excel 2016 version. The average score of all the participating laboratories and the average scores per section were calculated to assess their performance levels. Scores were converted into percentages. Student’s t test and the Kruskal‒Wallis test on the R version 4.3.2 software with a threshold of 5% were used to compare the scores of private and public laboratories.

The scores were generated per section, and the section scores were added together to make the overall performance score for the sentinel laboratory.

The averages of the scores per section and the overall scores of all the laboratories were calculated to assess the overall performance.

The “overall” or “section” performance was measured at three levels: “Satisfactory”, “Some improvement required” and “Significant improvement required”. The assessment levels, scores and criteria are summarized in Table 2.

**Table 2:** Scores of performance and appreciation level of sentinel laboratory

Score	Appreciation level	Comments
Overall or section score ≥ 80%	Satisfactory	The laboratory is registered for a quality program or it is formally involved a quality approach; the process is clearly defined, the objectives of the quality approach and their associated indicators are defined; procedures are developed and up to date; there is evidence of implementation of procedures.
Overall or section score between 40% and 80%	Some improvement required	The laboratory is registered for a quality program or is formally engaged in a quality approach; the process is defined, the procedures are developed but there is no evidence of implementation of the procedures; quality documents are not always up to date.
Overall or section score ≤ 39%	Significant improvement required	The laboratory is not registered for a quality program or there is no formal commitment to a quality approach. Process is not defined; procedures are missing and there is no evidence of implementation.

For ethical considerations, the anonymity of the AMR sentinel laboratories was kept through a Lab-Sx type coding system (where “Lab-S” stands for Sentinel Laboratory and “x” is a sequential numerical number).

### Communication of performance results to assessed sites

A template for the assessment report was developed and validated by the same technical committee. This report summarizes the scores of each section, the overall performance score of the participating laboratory, and the minimum and maximum scores of all participating laboratories. This report also highlighted the summary of strengths, areas for improvement and short-, medium- and long-term improvement actions. The individual site evaluation reports were approved by the head of the RNL-AMR and communicated by e-mail to the different sites within seven (7) days after the evaluation of the site. Each sentinel laboratory manager or respondent was asked for a return acknowledgment to confirm receipt of the assessment report.

## Results

### Profile of laboratories participating in the on-site assessment

All eighteen laboratories were evaluated. Among the public laboratories, eight were “national level” laboratories, including seven medical biology laboratories and one veterinary laboratory as per the hierarchy of the health pyramid (Table 3).

**Table 3:** Profile of the laboratories that participated in the EQA

N^o^	Name or Structure of belonging of the laboratory	Public/Private	Level in the Health pyramid	Region
1	CHR^a^ Koudougou	Public	Intermediate	Centre-Ouest
2	CHU^b^ Pediatric Charles De Gaulles (PCDG)	Public	National	Centre
3	CHU Yalgado Ouedraogo	Public	National	Centre
4	CHR Dedougou	Public	Intermediate	Boucle du Mouhoun
5	CHR Kaya	Public	Intermediate	Centre- Nord
6	CHR Gaoua	Public	Intermediate	Sud Ouest
7	Polyclinique SANDOF	Private	NC^c^	Centre
8	Polyclinique Notre Dame de la Paix (PCNDP)	Private	NC	Centre
9	Laboratoire national d’élevage (LNE)	Public	National	Centre
10	CHR de Banfora	Public	Intermediate	Cascades
11	CHU de Tengandogo	Public	National	Centre
12	Centre Muraz	Public	National	Hauts-Bassins
13	CHU de Bogodogo	Public	National	Centre
14	Laboratoire national de santé publique (LNSP)	Public	National	Centre
15	Hôpital Schiphra	Private	NC	Centre
16	CHU Souro SANOU	Public	National	Hauts-Bassins
17	CHR Tenkodogo	Public	Intermediate	Centre-Est
18	CHUR^d^ Ouahigouya	Public	Intermediate	Nord

^a^Regional Hospital Center; ^b^University Hospital Center; ^c^Not Classified; ^d^Regional University Hospital Center

### Overall performance of participating sentinel laboratories in the EQA

**Fig. 2:**
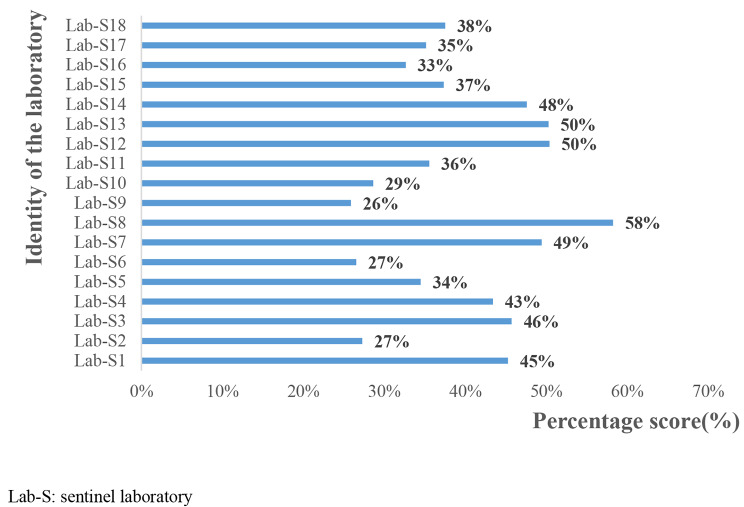
Overall performance of the 18 sentinel laboratories for AMR surveillance

The average overall performance score of all participants was 40%. The highest overall performance score was 58% and the lowest score was 26% (Fig. 2). No laboratory had a satisfactory overall score (≥ 80%); significant and some improvement was required for 55.5% and 45.5%, respectively.

### Performance scores by section of participating laboratories

**Fig. 3:**
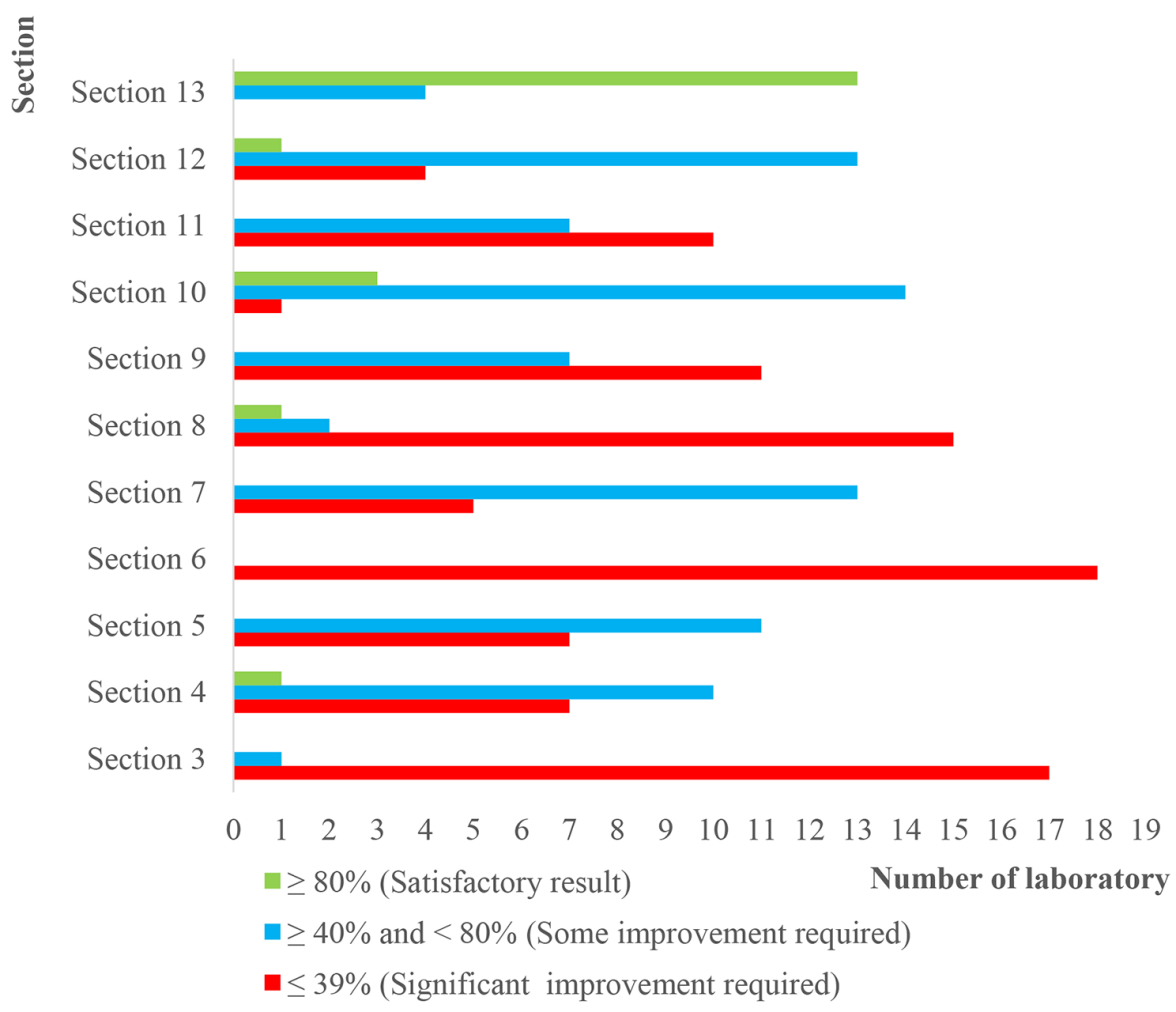
Distribution of the eighteen participating sentinel laboratories by category of section score

Section 11 “Storage of antibiotic discs” and Sect. 13 “Analytical steps for AST” were “not applicable” for one participating sentinel laboratory. The only section with more laboratories with a satisfactory score was the Sect. 13 with an average score of 85% for 76.5% (13/17) of the sentinel laboratories (Fig. 3).

**Fig. 4:**
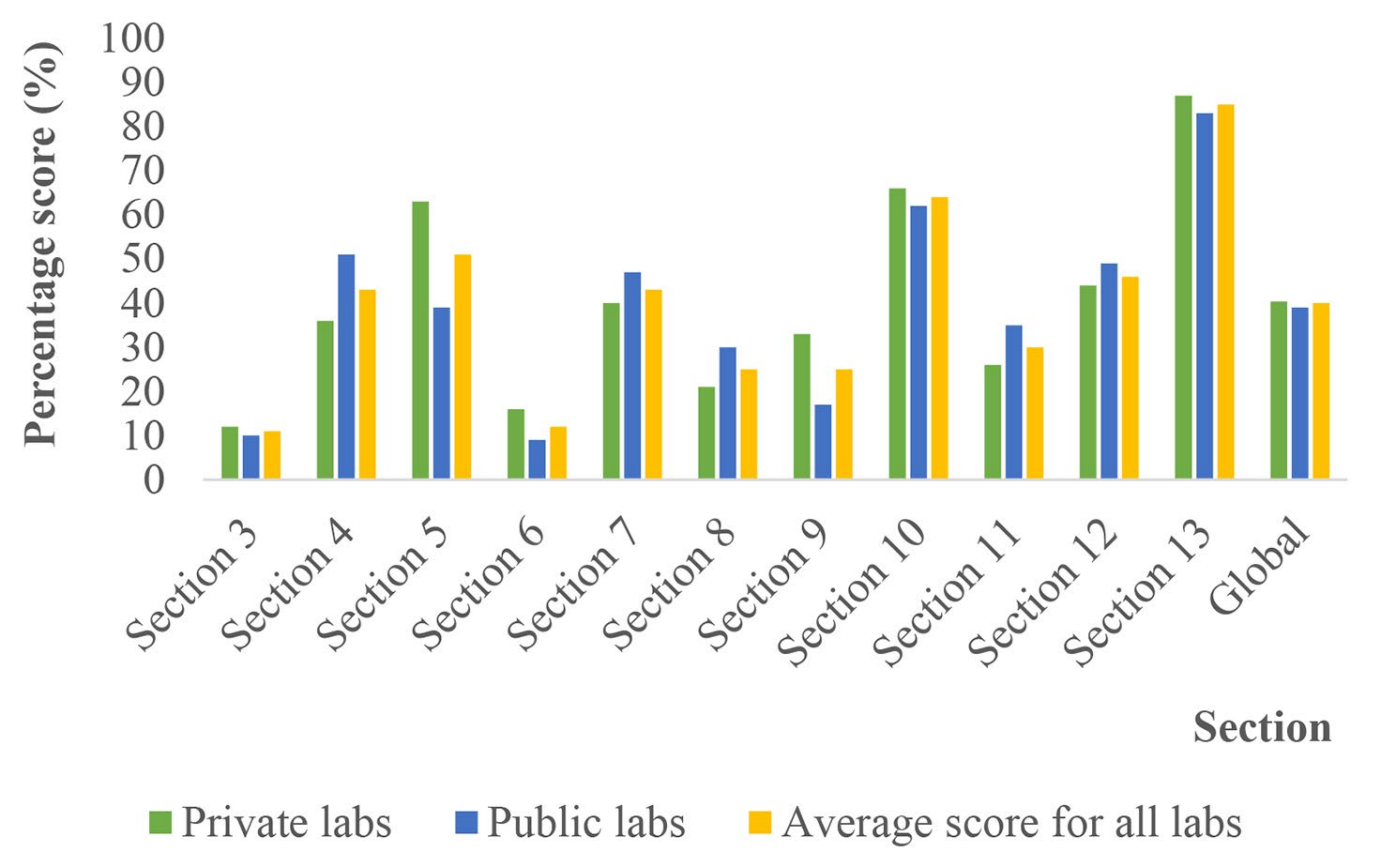
Average performance scores for private, public and all laboratories by section

Section 10 “EQA” had the 2nd best average score (64%). Some improvement was required for five Sects. (4, 5, 7, 10, 12), and significant improvement was required for five Sects. (3, 6, 8, 9, 11). The average overall scores were not significantly different between private and public laboratories (*p* value = 0.78) (Fig. 4).

## Discussion

This EQA in the form of on-site evaluation in the Sentinel laboratory-based antimicrobial resistance surveillance network in Burkina Faso is the first since the effective implementation of laboratory surveillance in 2018. One of the primary roles of the NRL-AMR is to carry out external quality assurance activities and monitor internal quality assurance, as was done in this study [3]. It is also original in the design of a checklist adapted to national requirements for quality assurance in the medical biology laboratory and to national procedures for carrying out AST. Despite the fact that this checklist takes into account international standards or directives, the originality of this checklist lies in the fact that it is based on the country’s requirements for antimicrobial susceptibility testing and the regulatory requirements in terms of quality. There are other checklists available, such as the AMR Laboratory Scorecard and the India Council for Medical Research (ICMR) AMR Checklists, developed as part of the AMR field assessment. Unfortunately, we were unable to consult some of these tools when preparing our checklist. The evaluation tool includes practical questions to improve the QMS and the technical aspects at the bench level, in particular the steps of AST. This evaluation tool could be adopted at the national level and used to assess, strengthen and support AMR sentinel site surveillance in the quality approach. The SLIPTA tool is an internationally recognized assessment tool, but it is very restrictive for the initial assessment of the QMS in the AMR sentinel sites in Burkina Faso. Laboratories with a score of 80% and above will be assessed in the next steps using the SLIPTA tool, in order to start the ISO 15,189 accreditation process.

The type of EQA chosen is the on-site evaluation because it allows us to give a realistic picture of sentinel laboratory practices by observing it under routine conditions to see if it meets quality requirements, to provide the information needed for internal improvement processes, to measure shortcomings and deficiencies, to know “where we are”, and to assist the laboratory in collecting the information necessary for planning, setting up training, monitoring and corrective actions [16].

The average overall performance score of participants shows that quality improvement actions are necessary for all participating laboratories. The Lab-S8 with the best performance score was a national level laboratory that implements ISO 15,189: 2012 standard [17] in its bacteriology section, and its staff participate in the quarterly supervision of AMR activities. This could justify the good quality performance score of this laboratory. In addition, 02 other laboratories (Lab-S12 and Lab-S13) stood out with an overall performance score of 50%. Indeed, Lab-S13 implements the ISO 15,189: 2012 standard [17] since 2019, and its staff have participated in quarterly supervision of AMR activities. Lab-S12 finds its strength by its staff, who are qualified and experienced, because the Lab-S12 staff participate in quarterly AMR supervision activities. The performance results of surveillance at these three AMR sentinel sites highlight the importance of the involvement of sentinel laboratory staff in quarterly supervision activities because participating as a supervisor allows them to gain experience from other sentinel laboratories. It would therefore be useful for the NRL to consider the staff of the eighteen sentinel laboratories to participate in quarterly supervisions.

These low scores reflect inadequate performance in the quality of sentinel AMR laboratories in Burkina Faso and may have an impact on the quality of AMR surveillance in Burkina Faso. In fact, unreliable surveillance data could have direct consequences on patient care at sentinel sites or on the epidemiology of AMR surveillance. Patient care will be affected by the administration of inappropriate treatment leading to complications, increased treatment costs and longer hospital stays. Concerning the consequences on the epidemiology of AMR surveillance, we may obtain an overestimation or underestimation of AMR frequencies at local, national level, which does not contribute effectively the updating of the list of essential antibiotics, the national treatment guidelines and the evaluation of the effects of the different treatment strategies put in place. This means motivating sentinel AMR laboratories to commit to implementing the corrective action plan. The government of Burkina Faso, for its part, could take the strong measures to support these laboratories in implementing an effective QMS.

For the section on the “Registration for a quality program and certification”, nine public laboratories had participated in a DLBM quality program Stepwise Laboratory quality Improvement Process Toward Accreditation (SLIPTA) between October 2019 and July 2021 [18]. The Lab-S8 was one of these nine laboratories and distinguished itself with at the end of the mentorship with two SLIPTA stars. One of the root causes of the common gaps is the lack of mentorship of all sentinel sites in quality assurance and the lack of proficiency testing (PT) by sending quality control strain panels. Another root cause is the fact that NRL in Burkina is not accredited. Efforts should be made to support the NRL toward accreditation so that its staff can support sentinel AMR surveillance sites. To establish the AMR surveillance network in Ethiopa, the mentorship system was introduced after the identification of sentinel sites to establish a one-year capacity building work plan for each site, and all mentored sites were enrolled in an EAQ program; the mentored sites also received quality control strains. This has brought satisfactory results, as by June 2018, the NRL in Ethiopia had achieved ISO 15189: 2012 international accreditation, and one additional site was preparing to be assessed for accreditation [19]. In Tanzania, all nine sentinel sites participate in external quality assurance, which is supported by the NHL and African Society for Laboratory Medicine [20]. Vounba et al. pointed out that the best way to strengthen the capacity of laboratories is to enroll them in the WHO Strengthening Laboratory Management Toward Accreditation (SLMTA) process [21]. This process allows a substantial improvement in the quality of laboratories, even if they do not complete the accreditation process. It would be important to enroll all sentinel laboratories in the AMR surveillance network in an SLMTA process.

For the section on the « Product and Materials », we essentially note the lack of a maintenance contract or program, list of equipment, metrological records and shortcomings in the management of reagents and consumables.

Regarding the section on the “Failure analysis, problem resolution and root cause analysis”, this study showed the absence of identification of non-conformities, the root causes analysis in case of failed quality control results of the culture media or antibiotics discs or any other problems found.

For the section on the “Management of staff competencies”, the non-conformities were related to the absence of staff competence assessment and staff records, in particular training certificates, diplomas and authorizations. The management of auxiliary equipment and reagents and consumables, the management of staff competencies and the management of non-conformities represent a challenge in the sentinel laboratories for the AMR surveillance network in Burkina Faso. These sections are less performant because they are very specific to the quality field, and sentinel laboratories have not received adequate training in quality assurance, as the other programm (HIV, tuberculosis); it will be necessary to organize training for managers or focal points of sentinel laboratories on the basics of QMS. In addition, it would be useful to support laboratory issues related to the metrology of critical auxiliary equipment (ovens, densitometer, PSM, etc.).

The only section with a satisfactory average score was compliance with the steps for AST. The assessment of the steps for AST by Katawa in 2021 in Togo gave an average compliance rate of 72.84% [8]. These results show the importance of standardization and the availability of manuals of procedures for performing AST in both countries. These strong points could be due to the benefits of regular training of laboratory staff on the technical aspects of AST in contrast to Tanzania, where few laboratory staff were well trained on AMR practices [20]. For example, an interuniversity diploma entitled AMR known in the French language as “Diplôme Inter Universitaire: Antibiologie et Antibiothérapie en Afrique Subsaharienne” is organized every year in Bobo-Dioulasso in Burkina Faso. This opportunity is offered to the personnel of the AMR sentinel laboratories for their participation.

The section with the 2nd best average score was the mastery of the principles of EQA by the AMR sentinel laboratories. AST is a critical point of the cytobacteriological examination requiring an EQA [22]. Sentinel laboratory staff are also made aware of the principles of EQA, and they regularly participate in the National Quality Control (NQC) program. We argue that continuous training on the technical aspects of AST and participation in the NQC program have a positive impact on the QMS performance of sentinel laboratories for AMR surveillance in Burkina Faso.

To improve their QMS, correctives actions plans were addressed to each laboratory individually, as each laboratory was not at the same level of quality performance. The outcomes of the assessment was shared with each laboratory along with proposed corrective actions to be implemented in short, medium and long term. In addition, correctives actions were addressed to the MoH authorities, AMR-NRL and DLBM to strengthen the QMS of sentinel laboratories and include:

- training AMR sentinel laboratories staff in the basics of QMS.
- inclusion of AMR sentinel laboratories in the SLMTA process.
- involvement of all staff of AMR sentinel laboratories in quarterly supervisions.
- introduction of a mentoring system to support these AMR sentinel laboratories in the quality approach.
- stimulating laboratories by informing them that those with a score of 80% and above will be able to move on to the next step by registering for the SLIPTA program, which will lead them to the ISO 15,189 accreditation process.

One of the major observations was the lack of AMR sentinel laboratory surveillance in the Sahel and eastern regions of Burkina Faso. This represents a major obstacle for AMR surveillance across the country because one of the strategies in the choice of sentinel laboratories was to consider their geographical distribution to cover the whole country [3]. Indeed, these two regions also face a major security challenge that impacts not only the AMR surveillance system but also healthcare services in general. One sentinel laboratory could not be evaluated on Sects. 11 and 13 of the checklist. Indeed, this laboratory already routinely used only the automated technique for AST, whereas these sections of the checklist were adapted for the disc diffusion method. In this study, one of the participating laboratories was a veterinary laboratory. This illustrates that the laboratory-based AMR surveillance network in Burkina Faso uses a “One Health” approach and responds to the recommendations of Moghnieh in 2019 on the need to put in place a multifaceted AMR containment programme based on the “One Health” approach [23].

## Conclusion

Quality performances were less satisfactory in all eighteen (18) AMR sentinel sites of Burkina Faso in this study. Corrective actions and recommendations were proposed to improve their quality approach. This activity constitutes a basic evaluation, and the mentorship of the AMR sentinel laboratory in the AMR surveillance network over the next three years would strengthen their QMS to guarantee the reliability of AST results and AMR prevalence in Burkina Faso.

## Data Availability

The completed collection tools for all eighteen participating sentinel laboratories, the evaluation tool, their individual report scores, the letter of authorization for the EQA from the MoH authorities and the terms of reference (TOR) are available from the corresponding author upon motivated request.

## Abbreviations

AMR: antimicrobial resistance
NRL: National Reference Laboratory
EQA: external quality assessment
NQC: national quality control
MoH: Ministry of Health
DLBM: Department of Medical Biology Laboratories
QMS: quality management system
AST: antimicrobial susceptibility test
